# Data describing the experimental design and quality control of RNA-Seq of human adipose-derived stem cells undergoing early adipogenesis and osteogenesis

**DOI:** 10.1016/j.dib.2019.105053

**Published:** 2019-12-31

**Authors:** Bruna H. Marcon, Lucia Spangenberg, Bernardo Bonilauri, Anny Waloski Robert, Addeli Bez Batti Angulski, Guillermo Cabrera Cabo, Axel R. Cofré, Paulo Sergio Loiacono Bettes, Bruno Dallagiovanna, Patrícia Shigunov

**Affiliations:** a Laboratory of Basic Biology of Stem Cells (LABCET), Instituto Carlos Chagas - FIOCRUZ-PR, Curitiba, Paraná, 81350-010, Brazil; b Unidad de Bioinformática, Institut Pasteur Montevideo, Mataojo 2020, Montevideo, 11400, Uruguay; c Cirurgia Plástica Dr. Paulo Bettes, Rua Francisco Rocha, 1312, Curitiba, PR, 80730-390, Brazil

**Keywords:** RNA-Seq, Human adipose-derived stem cells, Polysome profiling, Adipogenesis, Osteogenesis

## Abstract

An important tool to study the regulation of gene expression is the sequencing and the analysis of different RNA fractions: total, ribosome-free, monosomal and polysomal. By comparing these different populations, it is possible to identity which genes are differentially expressed and to get information on how transcriptional and translational regulation modulates cellular function. Therefore, we used this strategy to analyze the regulation of gene expression of human adipose-derived stem cells during the triggering of the adipogenic and osteogenic differentiation. Here, we have focused on analyzing the differential expression of mRNAs during early adipogenic and osteogenic differentiation, and presented the detailed data concerning the experimental design, the RNA-Seq quality data, the raw data obtained and the RT-qPCR validation data. This information is important to confirm the accuracy of the data considering a future reuse of the data provided. Moreover, this study may be used as groundwork for future characterization of the transcriptome and the translatome regulation of different cell types.

**Table:** Specifications Table

Subject	Biology
Specific subject area	Molecular Biology, Cell Biology, Adult Stem Cells, Transcriptome, Translatome
Type of data	Table Chart Graph Figure
How data were acquired	Sucrose density gradient elaboration (BioComp Model 108 Gradient Master ver. 5.3) Ultracentrifugation (HIMAC CP80WX, HITACHI) Fractionation of sucrose gradient (ISCO Model 160 Gradient Former Foxy Jr. Fraction Collector) RNA quality control (Agilent 2100 Bioanalyzer, Agilent) RNA-Seq (Illumina HiSeq 2500 System) Data analysis (Rsubread package; Bioconductor R package edgeR) RT-qPCR (SYBR® Green PCR Master Mix, Applied Biosystems®; LightCycler® 96, Roche)
Data format	Raw Analyzed
Parameters for data collection	Human adipose -derived stem cells (hASCs) were isolated from adipose tissue obtained from three healthy female donors that underwent liposuction surgery. Then hASCs were treated with maintenance (control – CT), adipogenic (ADI) or osteogenic (OST) induction medium for 24 h and submitted to total, ribosome-free, monosomal and polysomal RNA isolation.
Description of data collection	hASCs were kept in control medium or induced to adipogenesis or osteogenesis for 24 hours. The samples were submitted to sucrose density fractionation and the polysome profile was recorded to identify and to separate the ribosome-free, monosomal and polysomal RNA. Total RNA of each sample was also isolated. RNA-quality was checked and RNA-Seq were performed. The sequencing accuracy and the reproducibility of biological samples were also evaluated. The validation of RNA-Seq data was also performed by RT-qPCR.
Data source location	Institution: Instituto Carlos Chagas - FIOCRUZ-PR City/Town/Region: Curitiba, Paraná Country: Brazil
Data accessibility	Raw RNA-Seq data can be downloaded at the ArrayExpress repository under the ID E-MTAB-6298. Direct URL to data: https://www.ebi.ac.uk/arrayexpress/experiments/E-MTAB-6298/[2018]. Raw RT-qPCR dataset has been deposited in the Mendeley Data Repository: “DIB – RT-qPCR dataset”, Mendeley Data, https://data.mendeley.com/datasets/57bfj2z2kr/1
Related research article	A.W. Robert, A.B.B. Angulski, L. Spangenberg, P. Shigunov, I.T. Pereira, P·S.L. Bettes, H. Naya, A. Correa, B. Dallagiovanna, M.A. Stimamiglio, Gene expression analysis of human adipose tissue-derived stem cells during the initial steps of in vitro osteogenesis, Sci. Rep. 8 (2018) 4739. https://doi.org/10.1038/s41598-018-22991-6. B.H. Marcon, P. Shigunov, L. Spangenberg, I.T. Pereira, A.M. de Aguiar, R. Amorín, C.K. Rebelatto, A. Correa, B. Dallagiovanna, Cell cycle genes are downregulated after adipogenic triggering in human adipose tissue-derived stem cells by regulation of mRNA abundance, Sci. Rep. 9 (2019) 5611. https://doi.org/10.1038/s41598-019-42005-3.

**Table:** 

**Value of the Data** • The data provide information about total, ribosome-free, monosomal and polysomal RNA from hASCs during early adipogenesis and osteogenesis. • This dataset may be explored to understand the mechanisms involved in gene expression regulation involved in the balance between stemness and differentiation. • The information here provided may be used for future studies on triggering hASC differentiation processes. • The dataset can be used for direct comparison between adipogenesis and osteogenesis, or focused on the translational dynamic of individual transcripts, as well as studies about non-coding RNA expression and their interaction with the translational machinery.

## 1 Data

**Fig. 1:**
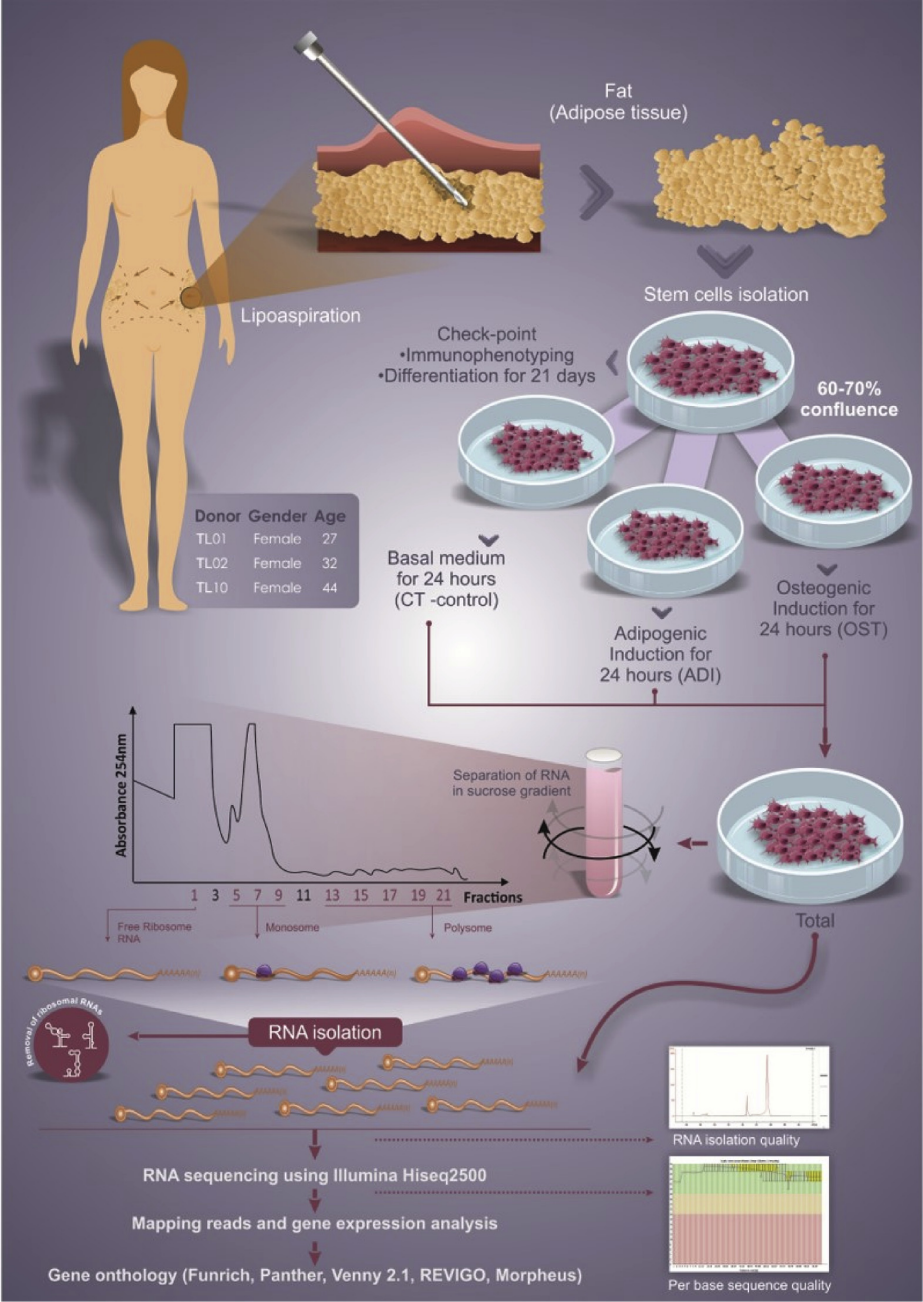
Workflow design for hASC isolation and collection for RNA-Seq analysis of total, ribosome-free, monosome-associated and polysome-associated RNAs.

In this report we show the data of a large-scale analysis by RNA-Seq of total, polysomal, monosomal and ribosome-free RNA fractions isolated from human adipose-derived stem cells (hASCs) after 24 hours of adipogenic or osteogenic induction. Here we focus on the process of RNA-Seq development, sequencing quality check, raw data obtainment and validation with RT-qPCR. The complete experimental design workflow is represented in Fig. 1.

**Fig. 2:**
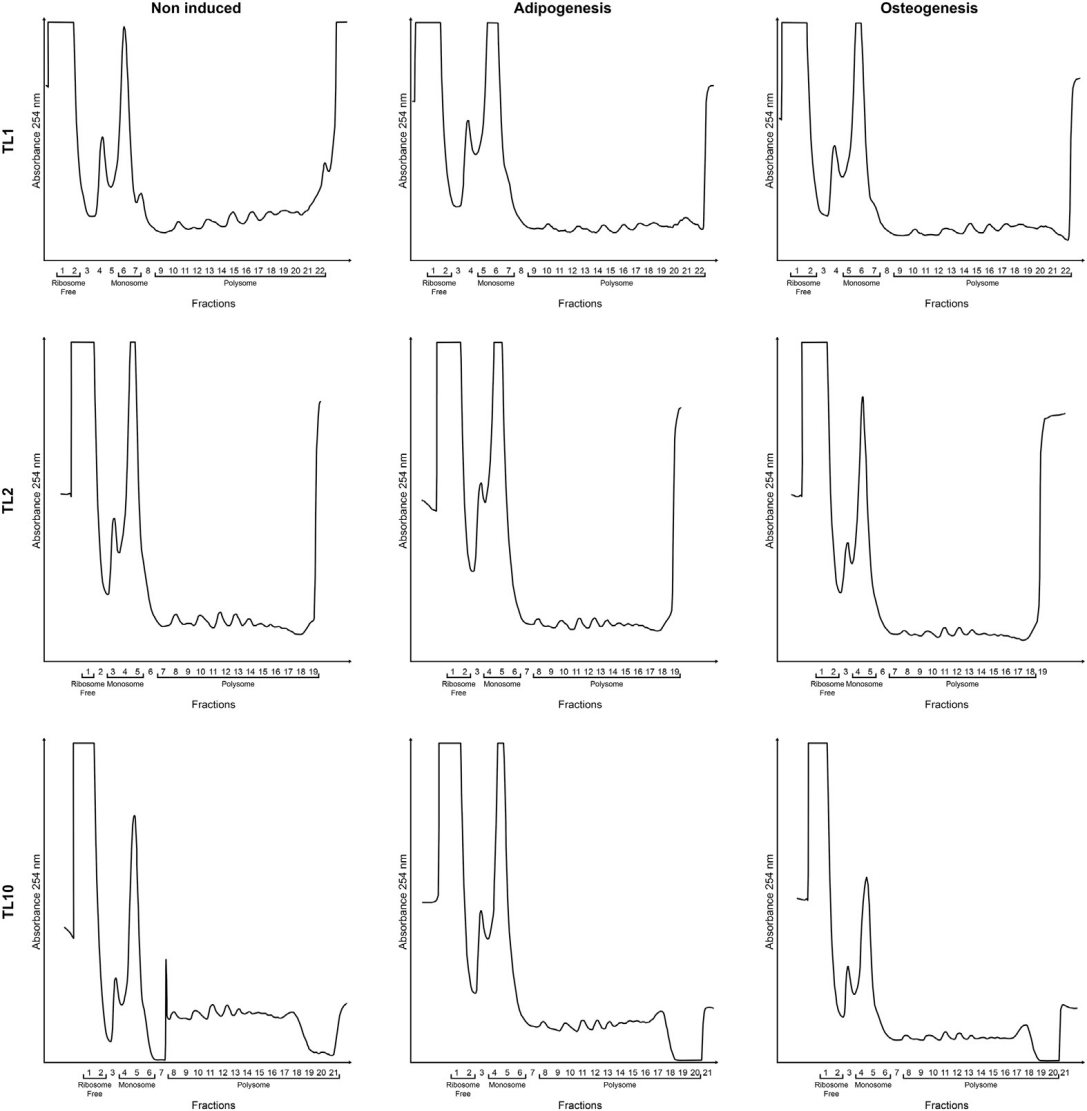
**Polysome profile obtained by sucrose gradient density fractionation.** hASCs isolated from 3 donors were treated with control (CT), adipogenic (ADI) and osteogenic (OST) induction media for 24 h and then submitted to sucrose gradient density fractionation. The polysome profile of each sample was recorded (absorbance at 254 nm). These are the full version with all polysome profiling graphics described in our related works [1,2]. Polysomes from TL10 CT and ADI [2], and TL2 CT and OST [1] were previously published.

**Fig. 3:**
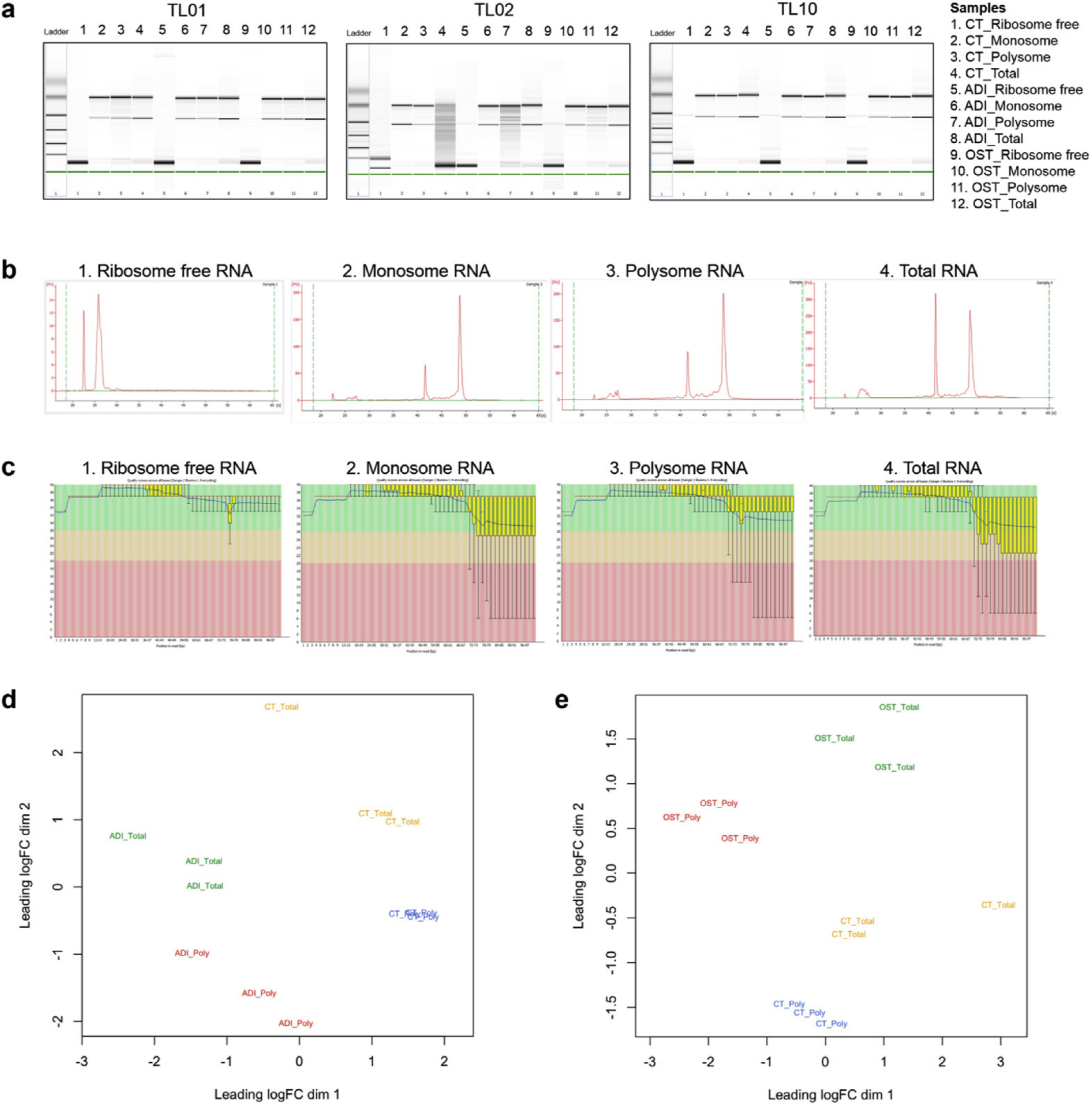
**RNA isolation quality data and per base sequence quality of RNA-sequencing reads.** (A) RNA quality of ribosome-free, monosome, polysome and total RNA from hASCs determined using the RNA 6000 Nano Chip. (B) Examples of electropherograms of ribosome-free, monosomal, polysomal and total RNA from hASCs as determined using the RNA 6000 Nano Chip. (C) Examples of per base sequence quality scores for ribosome-free, monosomal, polysomal and total RNA samples sequenced by FastQC (version 0.11.3). (D–E) MDS plot of polysomal and total RNA fractions for adipogenic (D) and osteogenic (E) differentiation. CT: control, hASCs treated with maintenance medium for 24 h; ADI: hASCs treated with adipogenic medium for 24 h; OST: hASCs treated with osteogenic medium for 24 h.

After isolation and characterization, the hASC were treated with control, adipogenic or osteogenic medium for 24 hours. The polysome profiles of hASCs (from the 3 donors) treated with different induction media obtained by sucrose density gradient are represented in Fig. 2. Using this approach, the fractions corresponding to ribosome-free, monosome-associated and polysome-associated RNAs could be separated for posterior RNA purification. Total RNA was also extracted. The quality and concentration of the isolated RNA were analyzed in order to determine their suitability for RNA-sequencing using the Agilent RNA 6000 Nano Kit and Agilent 2100 Bioanalyzer instrument. In Fig. 3A, the RNA quality is demonstrated based on the presence of 18S and 28S ribosomal RNA except for the ribosome-free RNA samples (samples 1, 5 and 9). This result is also shown in Fig. 3B with examples of electropherograms.

All samples were prepared for sequencing according to the TruSeq Stranded Total RNA manufacturer's manual. For the fragmentation step, the time of incubation was adjusted according to the RNA integrity number (RIN) (Fig. 3A). High-quality sequencing data were obtained as shown in Fig. 3C, which shows an example of sample from all RNA fractions. The quality distribution per read position is shown, revealing that most positions were of high quality along the entire read (more than half of the reads lengths had quality values higher than 35, and almost all the read lengths had quality values above 29). The same pattern was observed in all samples. Table 1 shows the number of reads obtained in each run (an average of ∼19,800,000 reads) and the numbers and percentages of mapped reads (average was roughly 82%). Raw RNA-Seq data as generated by Illumina Hiseq 2500 can be downloaded at the ArrayExpress repository under the ID E-MTAB-6298. This site serves as a landing page for this data and includes a description of the project, metadata and raw sequencing files (https://www.ebi.ac.uk/arrayexpress/experiments/E-MTAB-6298/[2018]). The log_2_ fold change (FC) values were calculated comparing ADI vs. CT and OST vs. CT in ribosome-free, monosomal, polysomal and total RNA. The differential expression analysis were published as supplementary material in previous articles [1,2].

**Table 1:** Reads obtained for each sample, number of reads mapped onto the genome and percentage of mapped reads. This information is the expanded version of brief descriptions presented in our previous works [1,2]. Note that sample “TL01_ADI_Monosome” has a lower number of mapped reads due to an overrepresentation of ribosomal genes.

Condition	Processed_reads	Mapped_reads	%mapped
TL01_CT_Total	8,822,585	6,194,842	70,22
TL01_CT_Free	19,443,837	15,943,455	82,00
TL01_CT_Monosome	23,333,095	15,363,306	65,84
TL01_CT_Polysome	19,649,163	14,917,916	75,92
TL01_ADI_Total	13,547,492	10,099,467	74,55
TL01_ADI_Free	12,234,077	5,935,227	48,51
TL01_ADI_Monosome	14,470,966	4,831,549	33,39
TL01_ADI_Polysome	17,658,467	12,238,539	69,31
TL01_OST_Total	17,167,026	12,421,820	72,36
TL01_OST_Free	12,818,330	10,756,365	83,91
TL01_OST_Monosome	14,434,613	10,910,730	75,59
TL01_OST_Polysome	30,457,820	24,111,460	79,16
TL02_CT_Total	22,269,425	20,320,186	91,25
TL02_CT_Free	24,190,750	20,816,597	86,05
TL02_CT_Monosome	17,404,225	13,350,410	76,71
TL02_CT_Polysome	18,461,466	16,376,781	88,71
TL02_ADI_Total	18,760,553	17,437,420	92,95
TL02_ADI_Free	14,861,502	13,120,676	88,29
TL02_ADI_Monosome	15,623,676	14,386,266	92,08
TL02_ADI_Polysome	18,476,485	17,139,154	92,76
TL02_OST_Total	15,180,100	14,108,666	92,94
TL02_OST_Free	12,583,722	11,101,517	88,22
TL02_OST_Monosome	13,887,007	12,702,981	91,47
TL02_OST_Polysome	31,811,986	28,796,870	90,52
TAL10_CT_Total	26,358,504	21,670,899	82,22
TAL10_CT_Free	15,484,134	13,471,362	87,00
TAL10_CT_Monosome	21,961,128	15,302,950	69,68
TAL10_CT_Polysome	16,299,496	14,057,869	86,25
TAL10_ADI_Total	17,673,116	15,436,770	87,35
TAL10_ADI_Free	21,632,960	18,745,157	86,65
TAL10_ADI_Monosome	22,657,003	15,744,744	69,49
TAL10_ADI_Polysome	25,469,357	20,803,234	81,68
TAL10_OST_Total	16,425,484	13,389,961	81,52
TAL10_OST_Free	4,292,457	3,739,582	87,12
TAL10_OST_Monosome	16,578,042	13,939,477	84,08
TAL10_OST_Polysome	19,652,104	16,838,969	85,69
Mean	19,869,615,4	16,380,940	81,74,031,229

Multidimensional scaling (MDS) plots were determined to assess the reproducibility of our data. For each differentiation (adipogenesis/osteogenesis) and for total and polysomal fractions, one MDS plot was performed. It was demonstrated that the pairs of RNA fractions were visualized together, e.g., polysomal and total RNA fractions for adipogenic (Fig. 3D) and osteogenic (Fig. 3E) differentiation. Four homogenous sample groups were observed: stem cell total RNA (orange), stem cell polysomal fraction (blue), induced total RNA (green) and induced polysomal fraction (red).

### 1.1 Data validation

**Fig. 4:**
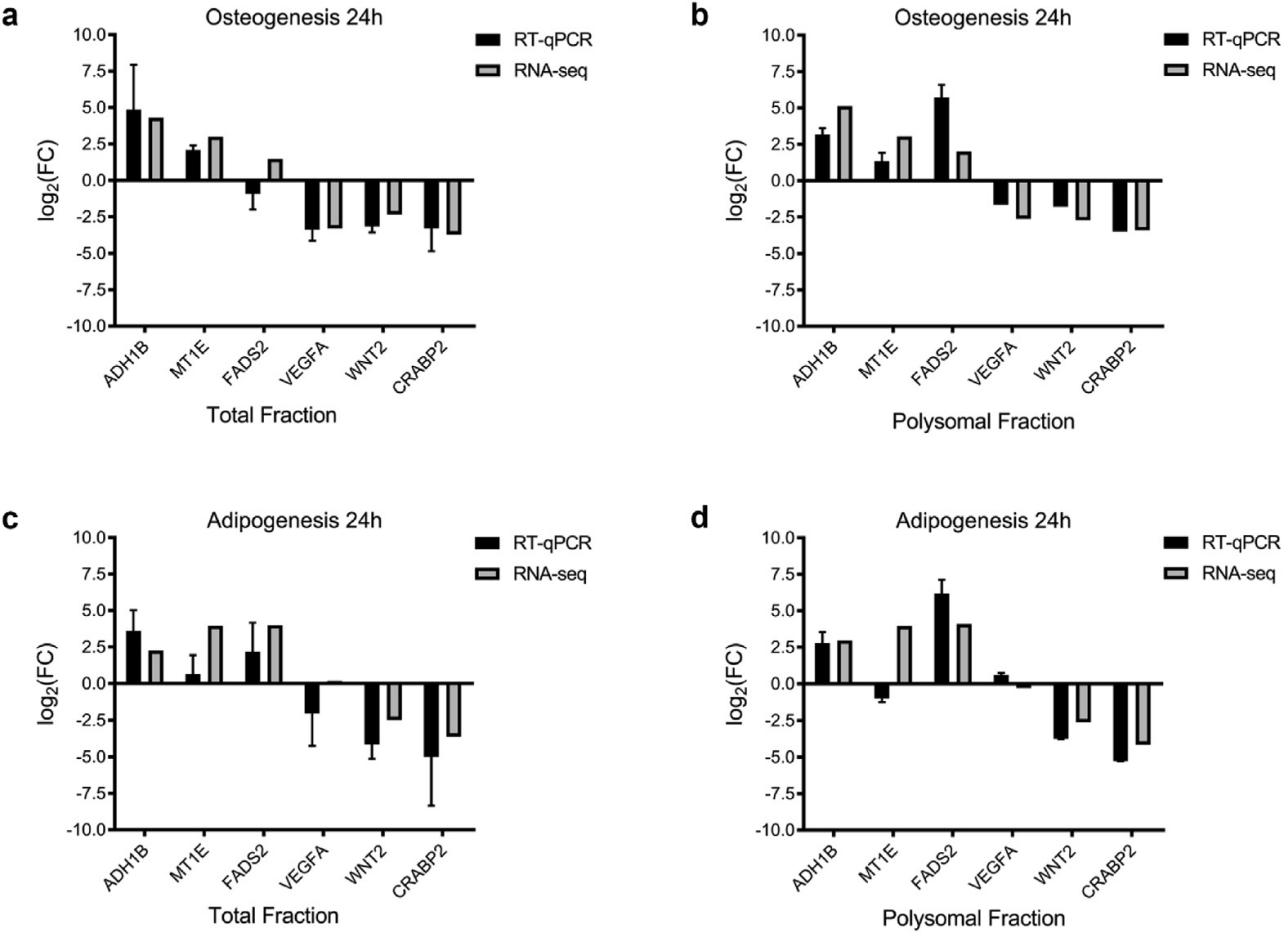
**RT-qPCR validation of six differentially expressed genes.** (A–B) Fold change differences in expression after osteogenic differentiation (OSTvsCT) in total and polysomal fractions comparing RT-qPCR and RNA-Seq. (C–D) Fold change differences in expression after adipogenic differentiation (ADIvsCT) in total and polysomal fraction comparing RT-qPCR and RNA-Seq. Fold change differences were calculated using log_2_ base values. RT-qPCR analysis of the total fraction was performed in technical and biological triplicate. RT-qPCR analysis of the polysomal fraction was performed in technical triplicate and biological duplicate. RNA-Seq analysis was performed in biological triplicate.

Validation of RNA-Seq was performed by quantitative RT-qPCR. First, we selected six genes previously identified as differentially expressed in the polysomal fraction of adipogenic [2] and osteogenic differentiation [1]. Expression values were normalized to fold changes for comparison. Although these two techniques are usually not comparable due to the utilization of different procedures, a high correlation was detected in our analysis. The similarities in the expression levels of these genes showed that our RNA-Seq data were consistent (Fig. 4).

We also compared our data with previously published studies [[3], [4], [5]] and, for example, observed that differentially expressed genes (DEGs) found by Ambele et al. [5] were also identified in our total and polysomal RNA-Seq fractions, with a similar profile (up or down regulated) in both experiments (Table 2). Notably, the methodologies used here (RNA-Seq) and by Ambele et al. (microarray) [5] provided information about overall population profiles during differentiation. Still, since hASCs are heterogeneous, other studies have focused on the analysis of intrapopulation differences in gene expression, using single-cell RNA-seq technology [6]. Further investigation using this technology in hASCs treated with induction media might identify different intrapopulation responses to these stimuli.

**Table 2:** Comparison of fold change of DEGs found by microarray (Ambele et al., 2016) and RNA-Seq analysis of hASCs induced to adipogenesis for 24 hours.

Gene Symbol	Ambele et al., 2016			Total		Poly	
	Fold Change (linear) (Induced vs. Control)	ANOVA p-value (Induced vs. Control)	FDR p-value	log (FC)	FDR	log (FC)	FDR
RGCC	120,79	0,000,043	0,021,075	9,583,771	7,4E-144	10,01,033,521	2,5E-158
PDK4	117,74	0,008,184	0,196,563	10,74,217	2,7E-137	8,81,757,307	1,7E-134
EDNRB	42,56	0,002,059	0,108,275	8,857,964	5,9E-116	10,32,436,544	2,5E-118
CPM	39,19	0,000,108	0,031,133	5,56,893	1,4E-77	4,91,979,404	4,9E-68
HSD11B1	33,09	0,000,026	0,018,656	8,381,996	6,21E-87	8,694,888,945	1,8E-105
MT1G	23,03	0,000,077	0,026,736	3,835,517	0,002207	4,195,654,196	1,21E-07
CRISPLD2	22,36	0,000,204	0,040,274	4,590,795	6,39E-35	4,859,236,086	7,1E-46
ALOX15B	21,68	0,000,002	0,00823	5,07437	0,000377	8,639,929,326	4,24E-12
RGS2	20,16	0,000,006	0,011,074	4,606,101	1,32E-82	4,619,559,121	1,77E-72
MT1M	18,13	0,000,007	0,011,546	4,810,601	5,15E-34	4,637,185,323	4,81E-22
NRCAM	15,79	0,000,003	0,008,747	4,224,598	7,56E-61	4,143,294,976	5,24E-53
GALNT15	14,38	0,001,332	0,088,615	3,370,781	2,05E-36	3,596,168,949	5,36E-32
METTL7A	14,17	0,000,335	0,048,518	4,043,354	3,56E-50	3,604,936,968	7,84E-40
RASD1	11,05	0,000,784	0,069,374	5,547,823	8,68E-33	6,325,800,774	3,22E-67
MAOA	11,02	0,003,463	0,137,229	6,964,571	1,49E-86	7,582,062,683	3E-99
SLC7A8	11,02	0,000,043	0,021,075	2,525,769	1,38E-10	3,414,536,268	1,74E-21
TMEM158	10,99	0,001,909	0,105,973	4,135,615	3,05E-11	5,365,494,547	3,27E-59
RASSF4	9,73	0,013,127	0,237,516	5,061,252	5,4E-36	4,96,042,219	8,79E-44
CNR1	8,93	0,011,758	0,227,199	8,273,254	9,56E-27	8,865,825,896	1,23E-41
IMPA2	8,79	0,000,438	0,052,725	3,204,121	1,19E-33	3,05,840,331	3,61E-36
MT1X	8,71	0,000,079	0,027,098	6,363,681	1,63E-22	5,437,885,251	6,15E-52
IFI44L	8,69	0,000,016	0,014,596	5,650,076	1,68E-47	4,336,312,458	6,67E-20
AOX1	7,48	0,000,093	0,028,629	3,396,809	1,76E-43	2,846,878,561	1,03E-34
CHRDL1	7,04	0,003,695	0,141,227	2,032,227	8,05E-06	1,924,777,838	1,21E-06
CCDC69	7,03	0,006,861	0,182,149	2,166,616	3,16E-09	2,844,188,223	2,26E-10
GREB1L	6,89	0,002,413	0,118,843	2,167,808	2,02E-18	2,498,854,887	7,07E-19
NEFL	6,87	0,00952	0,209,266	6,608,846	1,67E-61	6,487,955,633	8,54E-39
NEFM	6,8	0,035,527	0,352,753	0,594,523	0,766,164	0,80,997,685	0,458,129
LMO3	6,16	0,000,567	0,058,703	5,840,284	8,09E-93	6,675,000,848	8,8E-111
MT1F	6,06	0,037,603	0,360,309	4,109,284	0,003035	4,114,984,701	4,29E-05
PER1	5,92	0,000,064	0,024,908	3,146,617	3,76E-26	3,538,664,108	7,09E-33
SAMHD1	5,8	0,000,776	0,068,866	2,499,278	1,24E-19	2,709,463,657	1,23E-22
PDE7B	5,71	0,000,074	0,026,566	2,938,241	8,12E-17	3,654,733,538	8,85E-32
CIDEC	5,63	0,01131	0,224,821	7,62,812	4,08E-28	7,415,130,592	8,09E-38
FOXO1	5,56	0,000,431	0,052,725	3,252,448	9,86E-27	3,481,251,159	1,68E-20
IRS2	5,35	0,000,019	0,016,123	2,655,314	5,44E-11	2,658,758,457	2,66E-24
SAT1	5,25	0,000,011	0,013,102	2,670,537	3,94E-11	3,093,685,397	3,17E-28
RNF144A	5,06	0,000,884	0,074,754	4,520,335	5,62E-15	4,770,149,744	1,13E-13
REV3L	5,05	0,00008	0,02719	2,55,853	3,27E-25	2,413,279,429	1,06E-28
IL1R1	5,04	0,000,029	0,019,043	1,963,723	2,12E-18	2,170,891,211	3,75E-21
SLC19A2	4,97	0,00066	0,063,288	2,722,488	4,2E-14	3,27,026,976	1,35E-39
C10orf10	4,93	0,013,379	0,239,323	2,321,615	5,45E-17	2,371,303,595	5,8E-26
NID1	4,87	0,003,368	0,135,243	2,025,193	7,76E-16	2,358,309,296	1,01E-18
KLF15	4,76	0,004,771	0,157,798	4,807,511	5,3E-27	5,175,157,422	2,87E-36
FKBP5	4,7	0,000,184	0,039,103	4,412,921	4,86E-48	4,385,642,188	2,8E-34
GLUL	4,66	0,000,013	0,014,085	2,441,417	8,79E-17	2,130,908,436	4,36E-15
PRUNE2	4,59	0,001,501	0,093,669	2,863,764	3,18E-28	2,049,045,765	1,87E-11
FASN	4,56	0,000,154	0,036,445	2,238,049	1,18E-16	2,64,236,721	1,45E-23
ANGPTL1	4,38	0,012,054	0,229,591	3,460,774	4,43E-27	3,112,552,243	8,43E-34
WNT5A	4,38	0,002703	0,124,034	1,844,243	1,22E-09	2,01,640,862	1,83E-15
PIK3C2B	4,37	0,006,708	0,18,066	4,55,401	4,42E-09	2,9,757,817	0,000872
TSC22D3	4,32	0,000,151	0,036,125	3,638,564	5,31E-42	3,841,132,005	1,71E-44
CDH20	4,21	0,000,393	0,050,838	8,486,391	3,51E-10	8,13,813,717	4,76E-10
HIP1	4,21	0,000,053	0,022,892	1,533,995	2,02E-08	1,291,126,204	0,001741
ZBTB16	4,18	0,000,087	0,027,826	9,040,063	1,34E-78	9,300,261,657	3,45E-76
MME	4,16	0,00024	0,041,932	2,142,862	1,47E-14	1,891,072,886	1,68E-10
GAS1	4,11	0,000,407	0,051,911	3,337,304	1,12E-46	2,98,235,324	4,35E-42
MT1E	4,05	0,00125	0,086,384	3,954,136	6,94E-22	3,953,500,629	2,31E-68
TMTC1	4,01	0,006,396	0,177,376	1,1612	1,81E-05	1,133,840,503	0,000267
ADAMTSL1	−4,03	0,000,168	0,038,063	−1,37,591	7,8E-11	−1,26,694,561	7,65E-07
ITGA3	−4,1	0,018,247	0,271,424	−2,65,895	1,53E-14	−2,51,334,663	6,3E-13
E2F7	−4,12	0,00039	0,050,838	−2,84,783	1,28E-22	−2,93,986,447	6,75E-22
GINS2	−4,15	0,021,165	0,288,914	−2,36,118	2,05E-09	−2,33,010,649	1,75E-13
DUSP14	−4,15	0,000,099	0,029,542	−1,34,825	6,44E-07	−1,62,871,791	4,99E-13
ICAM1	−4,2	0,002,545	0,121,542	−0,42,473	0,730,455	−0,48,762,359	0,544,911
SMURF2	−4,23	0,000,462	0,053,322	−2,27,944	5,38E-26	−2,24,431,984	6,32E-24
FAM129A	−4,24	0,000,188	0,039,211	−1,46,394	7,42E-12	−1,20,811,435	0,00089
FGL2	−4,31	0,019,915	0,28,208	0,282,329	0,514,765	0,017,445,377	0,968,386
HSPB7	−4,44	0,010,967	0,221,978	−1,9817	2,47E-18	−1,98,497,474	2,17E-15
FMN2	−4,45	0,000,054	0,022,892	−2,96,442	6,13E-28	−3,5,027,012	1,78E-50
FGF5	−4,46	0,001,809	0,104,171	−4,24,254	1,49E-18	−3,60,650,567	3,61E-26
SGOL1	−4,59	0,006,871	0,182,149	−3503	0,000169	−2,5,583,294	2,97E-11
ANKRD1	−4,6	0,001,819	0,104,308	−1,87,855	1,26E-14	−1,91,012,655	1,09E-18
NTN4	−4,69	0,007,655	0,1903	−1,59,132	1,39E-07	−1,53,017,104	3,07E-08
DRAM1	−4,72	0,000,466	0,053,448	−0,31,532	0,414,653	−0,27,518,891	0,387,709
SMAGP	−4,73	0,00536	0,164,354	−2,99,995	1,35E-06	−3,19,063,126	1,21E-12
GDF15	−4,75	0,0028	0,126,143	−0,6388	0,273,194	−0,55,899,591	0,15,433
SEMA3D	−4,78	0,043,316	0,379,719	−2,47,423	3,65E-10	−2,8,541,466	1,29E-24
TBC1D2	−4,83	0,000,023	0,01717	−3,31,394	2,36E-31	−2,68,770,313	2,42E-21
SKA1	−4,89	0,001,783	0,103,744	−2,2233	1,29E-15	−2,56,280,893	2,67E-22
GPR133	−4,92	0,00305	0,130,327	−2,10,295	4,18E-09	−1,79,272,973	6,46E-08
FGF2	−5,01	0,000,336	0,048,518	−3,23,847	1,06E-22	−3,2,984,631	8,5E-33
RAB3B	−5,06	0,00006	0,024,259	−2,89,455	1,61E-33	−2,71,126,656	1,06E-35
DACT1	−5,21	0,000,068	0,025,539	−1,93,568	2,25E-09	−1,78,266,976	1,46E-09
SLC9A7	−5,24	0,003,136	0,131,871	−2,76,362	1,94E-16	−2,12,515,742	6,67E-14
GALNT12	−5,24	0,005,206	0,162,365	−2,87,133	8,25E-10	−1,49,336,491	0,000391
FST	−5,29	0,000,846	0,072,981	−2,12,037	1,85E-13	−2,12,003,115	1,02E-09
NEK7	−5,34	0,000,151	0,036,125	−2,26,857	6,06E-19	−2,72,554,749	5,29E-20
IER3	−5,36	0,000,014	0,014,085	−2,48,028	2,14E-17	−2,86,733,609	1,58E-26
MYBL1	−5,37	0,005,019	0,160,736	−2,5528	1,86E-18	−2,67,503,786	4,47E-26
DOK5	−5,48	0,000,028	0,019,043	−2,13,303	2,38E-16	−2,13,132,492	1,55E-17
LPXN	−5,69	0,004,656	0,155,542	−1,36,606	0,016,002	−2,99,973,926	1,32E-14
DIAPH3	−5,79	0,000,148	0,035,982	−3,13,824	1,51E-30	−2,83,385,136	1,4E-22
KCND2	−5,94	0,005,904	0,171,879	−3,27,698	2,53E-31	−2,93,320,861	3,63E-22
GREM2	−6,01	0,000,744	0,067,469	−2,41,739	5,53E-26	−2,25,181,116	6,13E-23
ARNTL2	−6,12	0,002,989	0,128,943	−2,32,456	1,76E-12	−2,07,141,405	9,66E-12
PTPRB	−6,2	0,000,175	0,038,908	−4,97,634	3,37E-58	−4,47,132,516	2,53E-42
MMP1	−6,25	0,00034	0,048,586	−3,12,988	1,87E-10	−3,47,944,063	6,86E-08
ALPK2	−6,33	0,000,224	0,041,323	−2,47,245	2,1E-26	−2,69,629	6,69E-37
KLF2	−6,49	0,000,046	0,021,075	−3,27,652	8,57E-07	−3,96,887,126	3,55E-20
TM4SF1	−6,61	0,019,561	0,279,336	−1846	0,001055	−1,52,614,968	0,044,901
MYCT1	−6,85	0,000,000,147	0,003227	−8,08559	3,41E-07	−6,6,405,274	2,97E-18
NOV	−7,04	0,000,634	0,062,021	−3,82,171	4,37E-18	−3,73,758,601	1,82E-28
OXTR	−7,22	0,001,265	0,086,384	−5,53,372	8,61E-27	−5,43,323,833	6,06E-34
TEK	−7,37	0,000,000,136	0,003227	−2,45,975	2,53E-21	−2,16,703,129	1,62E-23
SLC4A4	−8	0,000,035	0,020,495	−3,04828	9,41E-24	−3,07,718,419	4,79E-18
PDE1C	−8	0,00061	0,061,035	−2,94,725	1,92E-35	−3,25,952,741	7,71E-46
KRT18P10	−8,11	0,000,239	0,041,932	−4,32,262	4,06E-18	−3,77,414,763	2,25E-08
SCN9A	−8,28	0,003,494	0,137,508	−2,85,813	3,25E-14	−3,18,676,756	4,21E-20
RHOJ	−8,29	0,000,014	0,014,085	−3,25,494	8,47E-12	−2,98,252,297	1,58E-13
NGF	−8,68	0,000,000,913	0,006291	−2,70,064	1,24E-14	−2,6,218,191	4,91E-23
CPED1	−8,9	0,000018	0,01575	−2,45,922	2,92E-14	−2,52,098,464	4,72E-18
ESM1	−9	0,00608	0,174,056	−6,04337	2,62E-17	−6,75,845,961	3,97E-41
CAP2	−9,05	0,000,023	0,01717	−2,97,654	8,76E-12	−3,47,311,237	9,98E-21
CH25H	−11,61	0,000,006	0,011,074	−0,7949	0,308,795	−0,85,740,999	0,105,523
IL6	−13,75	0,000,187	0,039,211	−2,16,551	1,26E-07	−2,92,639,867	3,22E-15
ATP8B1	−14,17	0,000,007	0,011,546	−3,06986	3,42E-27	−2,87,413,254	5,17E-29
TNFRSF11B	−15,1	0,000,004	0,010,118	−4,23,333	5,34E-55	−4,58,516,491	7,48E-74
KRT34	−15,65	0,000,333	0,048,472	−7,0997	1,45E-43	−6,83,306,019	2,38E-34
B3GALT2	−19,75	0,000,009	0,011,546	−3,49,733	2,57E-12	−4,30,658,287	1,12E-49

## 2 Experimental design, materials, and methods

### 2.1 Isolation, characterization, culture and differentiation of human hASC

hASCs were isolated from adipose tissue obtained from three healthy female donors (Table 3) that underwent liposuction surgery. This data was performed in accordance with the guidelines for research involving human subjects and with approval from the Ethics Committee of Fundação Oswaldo Cruz, Brazil (CAAE: 48,374,715.8.0000.5248). Informed consent was obtained from each donor. hASCs were isolated, characterized and cultivated as previously described [1,2]. These methods are expanded versions of descriptions in our previous work [1,2]. First, 200 mL of adipose tissue was washed with 1 L of sterile phosphate-buffered saline (PBS) (Gibco Invitrogen®, Carlsbad, CA, USA) and digested with 1 mg/mL collagenase type I (Gibco Invitrogen®, Carlsbad, CA, USA) diluted in PBS for 30 min at 37 °C and 5% CO_2_ under constant shaking. After the incubation period, the shaking was halted, and the cell suspension was allowed to stand for 5 minutes to separate the lipid-enriched phase (upper). The bottom phase was collected and filtered through a 100-μm mesh filter (BD Bioscience). The cell suspension obtained was centrifuged (10 min, 950×*g*, 8 °C), and the supernatant was discarded. The cell pellet was resuspended and treated with hemolysis buffer (0.83% ammonium chloride, 0.1% sodium bicarbonate and 0.04% EDTA) for 10 min to remove erythrocytes. After centrifugation (150×*g*, 10 min, 8 °C), the supernatant was discarded, and the cell pellet was resuspended in PBS and filtered through a 40-μm mesh filter (BD Bioscience). After centrifugation (350×*g*, 10 min, 8 °C), the supernatant was discarded, and the cells were plated at a density of 1 × 10^5^ cells/cm^2^ in T75 culture flasks in DMEM supplemented with 10% fetal bovine serum (FBS), penicillin (100 units/ml) and streptomycin (100 μg/ml). The flasks were incubated in a humidified incubator at 37 °C and 5% CO_2_. The culture medium was changed twice a week until the hASC cultures were 80–90% confluent, at which point the cells were trypsinized and expanded. All tests were performed with cell passaged 4 to 6 times.

**Table 3:** Donors of adipose tissue used in this data.

Donor	Sex	Age (years)	Weight (kg)	Height (m)	BMI (kg/m^2^)
TL01	Female	27	70.9	1.62	27.0157
TL02	Female	32	55	1.63	20.70,082
TL10	Female	44	69	1.71	23.597

Cell characterization was performed according to the minimal criteria for defining mesenchymal stem cells as established by the International Society for Cellular Therapy. First, cells were detached using trypsin-EDTA and incubated in blocking solution (1% bovine serum albumin - BSA - diluted in PBS) at 4 °C for 1 h. The cells were then incubated for one more hour at 4 °C in the dark with the following antibodies (diluted in blocking solution): FITC-conjugated anti-human CD90 (Thy1), CD34, CD31 and CD19; APC-conjugated anti-human CD73; and PE-conjugated anti-human CD45, HLA-DR, CD117 and CD11b. Mouse IgG antibodies (FITC, APC, PE) were used as negative controls. After incubation, the cells were washed once with PBS, and the data were acquired on a FACSCanto II instrument (Becton Dickinson). For each sample, at least 10,000 events were collected and analyzed with FlowJo® v.10 software (Flowjo, LLC).

For adipogenic and osteogenic differentiation, hASCs were treated with hMSC Adipogenic Differentiation Medium (hMSC Adipogenic BulletKit, Lonza) or hMSC Osteogenic Differentiation Medium (hMSC Osteogenic BulletKit, Lonza), respectively, according to the manufacturer's instructions. For the RNA-Seq analysis, cells were treated with maintenance (control – CT), adipogenic or osteogenic induction medium for 24 h. To assess the differentiation potential of the isolated hASCs, the cells were submitted to a longer induction treatment. Adipogenic differentiation was induced by cycles of treatment for 3 days with induction medium and 3–4 days of maintenance over a total of 28 days. The induction medium consisted of basal medium plus the adipogenic inducers indomethacin, insulin, dexamethasone and IBMX. Osteogenic differentiation was induced with medium containing β-glycerophosphate, ascorbic acid and dexamethasone over 21 days. The medium was replaced every 3–4 days. The efficiencies of adipogenic and osteogenic differentiation were determined by assessing the cytoplasmic accumulation of triglycerides with AdipoRed™ Assay Reagent (Lonza) or the mineralized extracellular matrix using the OsteoImage™ Mineralization Assay (Lonza), respectively.

### 2.2 Sucrose density gradient separation and RNA purification

Polysomal fractions were prepared as previously described [1,2]. These methods are expanded versions of descriptions in our related work [1,2]. First, hASC cultures at 60–70% of confluence were either induced to osteogenic (OST) or adipogenic (ADI) differentiation or kept in maintenance medium (control - CT) for 24 h. The cells were then treated with 0.1 mg/ml cycloheximide (Sigma Aldrich - St. Louis, MO, EUA) diluted in culture medium for 10 min at 37 °C. Cells were detached with trypsin and then centrifuged (700×*g*, 5 min, 8 °C), and the resulting cell pellets were washed twice in 0.1 mg/ml cycloheximide diluted in PBS. After centrifugation (700×*g*, 5 min, 8 °C), the cell pellet was resuspended in lysis buffer (15 mM Tris HCl (pH 7.4), 15 mM MgCl_2_, 300 mM NaCl, 100 μg/mL cycloheximide, 1% Triton X-100) and incubated for 10 min on ice. The cell lysates were centrifuged (12,000×*g*, 10 min, 4 °C), and the supernatants were carefully isolated and loaded onto 10–50% sucrose gradients (BioComp Model 108 Gradient Master ver. 5.3). Gradients were subjected to ultracentrifugation (150,000×*g*, SW40 rotor, HIMAC CP80WX HITACHI, 160 min, 4 °C) and then fractionated with the ISCO gradient fractionation system (ISCO Model 160 Gradient Former Foxy Jr. Fraction Collector) connected to a UV detector to monitor absorbance at 254 nm. The polysome profile was recorded.

The ribosome-free, monosome-associated and polysome-associated RNA fractions as well as the total RNA were extracted using the Direct-zol™ RNA MiniPrep kit (Zymo Research) according to the manufacturer's instructions.

### 2.3 cDNA library construction

In total, 1 μg of RNA from three independent biological sample replicates of each condition (ribosome-free, monosome-associated, polysome-associated and total RNA) was used for cDNA library construction and RNA-Seq (Table 4). The cDNA libraries were prepared with the TruSeq Stranded Total RNA Sample Preparation kit (Illumina, Inc.) following the manufacturer's instructions. The library size was verified using the Agilent 2100 Bioanalyzer (Agilent), and the library concentration was confirmed by qPCR using the Illumina Library Quantification Kit Universal qPCR mix (Kapa Biosystems).

**Table 4:** Description of data generated from the RNA-sequencing analysis of individually collected total, free, monosomal and polysomal cells deposited in ArrayExpress under the number E-MTAB-6298.

Sample	Organism	Organism Part	Cell Type	Material Type	Fraction	Differentiation induction	Donor
**TL01_ADI_Free**	Homo sapiens	Adipose tissue	ASC	RNA	Free	Adipogenesis	TL01
**TL01_ADI_Mono**	Homo sapiens	Adipose tissue	ASC	RNA	Monosome	Adipogenesis	TL01
**TL01_ADI_Poly**	Homo sapiens	Adipose tissue	ASC	RNA	Polysome	Adipogenesis	TL01
**TL01_ADI_Total**	Homo sapiens	Adipose tissue	ASC	RNA	Total	Adipogenesis	TL01
**TL01_CT_Free**	Homo sapiens	Adipose tissue	ASC	RNA	Free	Control - Non-differentiated	TL01
**TL01_CT_Mono**	Homo sapiens	Adipose tissue	ASC	RNA	Monosome	Control - Non-differentiated	TL01
**TL01_CT_Poly**	Homo sapiens	Adipose tissue	ASC	RNA	Polysome	Control - Non-differentiated	TL01
**TL01_CT_Total**	Homo sapiens	Adipose tissue	ASC	RNA	Total	Control - Non-differentiated	TL01
**TL01_OST_Free**	Homo sapiens	Adipose tissue	ASC	RNA	Free	Osteogenesis	TL01
**TL01_OST_Mono**	Homo sapiens	Adipose tissue	ASC	RNA	Monosome	Osteogenesis	TL01
**TL01_OST_Poly**	Homo sapiens	Adipose tissue	ASC	RNA	Polysome	Osteogenesis	TL01
**TL01_OST_Total**	Homo sapiens	Adipose tissue	ASC	RNA	Total	Osteogenesis	TL01
**TL02_ADI_Free**	Homo sapiens	Adipose tissue	ASC	RNA	Free	Adipogenesis	TL02
**TL02_ADI_Mono**	Homo sapiens	Adipose tissue	ASC	RNA	Monosome	Adipogenesis	TL02
**TL02_ADI_Poly**	Homo sapiens	Adipose tissue	ASC	RNA	Polysome	Adipogenesis	TL02
**TL02_ADI_Total**	Homo sapiens	Adipose tissue	ASC	RNA	Total	Adipogenesis	TL02
**TL02_CT_Free**	Homo sapiens	Adipose tissue	ASC	RNA	Free	Control - Non-differentiated	TL02
**TL02_CT_Mono**	Homo sapiens	Adipose tissue	ASC	RNA	Monosome	Control - Non-differentiated	TL02
**TL02_CT_Poly**	Homo sapiens	Adipose tissue	ASC	RNA	Polysome	Control - Non-differentiated	TL02
**TL02_CT_Total**	Homo sapiens	Adipose tissue	ASC	RNA	Total	Control - Non-differentiated	TL02
**TL02_OST_Free**	Homo sapiens	Adipose tissue	ASC	RNA	Free	Osteogenesis	TL02
**TL02_OST_Mono**	Homo sapiens	Adipose tissue	ASC	RNA	Monosome	Osteogenesis	TL02
**TL02_OST_Poly**	Homo sapiens	Adipose tissue	ASC	RNA	Polysome	Osteogenesis	TL02
**TL02_OST_Total**	Homo sapiens	Adipose tissue	ASC	RNA	Total	Osteogenesis	TL02
**TL10_ADI_Free**	Homo sapiens	Adipose tissue	ASC	RNA	Free	Adipogenesis	TL10
**TL10_ADI_Mono**	Homo sapiens	Adipose tissue	ASC	RNA	Monosome	Adipogenesis	TL10
**TL10_ADI_Poly**	Homo sapiens	Adipose tissue	ASC	RNA	Polysome	Adipogenesis	TL10
**TL10_ADI_Total**	Homo sapiens	Adipose tissue	ASC	RNA	Total	Adipogenesis	TL10
**TL10_CT_Free**	Homo sapiens	Adipose tissue	ASC	RNA	Free	Control - Non-differentiated	TL10
**TL10_CT_Mono**	Homo sapiens	Adipose tissue	ASC	RNA	Monosome	Control - Non-differentiated	TL10
**TL10_CT_Poly**	Homo sapiens	Adipose tissue	ASC	RNA	Polysome	Control - Non-differentiated	TL10
**TL10_CT_Total**	Homo sapiens	Adipose tissue	ASC	RNA	Total	Control - Non-differentiated	TL10
**TL10_OST_Free**	Homo sapiens	Adipose tissue	ASC	RNA	Free	Osteogenesis	TL10
**TL10_OST_Mono**	Homo sapiens	Adipose tissue	ASC	RNA	Monosome	Osteogenesis	TL10
**TL10_OST_Poly**	Homo sapiens	Adipose tissue	ASC	RNA	Polysome	Osteogenesis	TL10
**TL10_OST_Total**	Homo sapiens	Adipose tissue	ASC	RNA	Total	Osteogenesis	TL10

### 2.4 Large-scale sequencing

The samples were prepared for sequencing on the Illumina Platform using the TruSeq Stranded Total RNA LT Kit. For clustering and sequencing, the TruSeq SR Cluster Kit v3 - cBot – HS and TruSeq SBS Kit v3 - HS (100-cycles) were used. Samples were sequenced on the Illumina HiSeq 2500 System. The raw data were deposited in ArrayExpress under the number E-MTAB-6298.

### 2.5 Bioinformatic analyses

Sequence data were mapped and counted by comparison against the latest version of the GRCh38 human genome with the Rsubread package. The mapping of reads was done with default parameters (unique mapping of reads), and counting was performed using the Ensembl annotation (GRCh38).

For quality evaluation purposes, we performed multidimensional analysis (MDS; multidimensional scaling), a method involving dimension reduction of the count matrix, to explore associations between variables. The log-2 transformation values of the raw counts were used for this analysis, and rows with no information were eliminated (0 counts in all samples). Samples of the same condition should cluster together to ensure consistency and replicability of the data.

For comparisons of gene expression between samples, RPKM values (reads per kilobase per million mapped reads) were determined. Differential expression analysis was performed using the Bioconductor R package edgeR [7]. Different comparisons were considered for adipogenesis and osteogenesis. For each RNA fraction (polysomal, monosomal, total and ribosome-free RNA), the induced condition (ADI or OST) versus the stem cell state (CT - control) was analyzed. This analysis included genes with at least one count per million in at least three samples. After a normalization procedure using three recommended methods (estimateGLMCommonDisp, estimateGLMTrendedDisp, estimateGLMTagwiseDisp), differential expression analyses of all comparisons were performed using the generalized linear mixed model (glmFit and glmLRT). Correction for multiple testing was performed with the FDR (false discovery rate). The data from some of these analyses with the parameters mentioned above are shown in Robert et al., 2018 [1], Marcon et al., 2019 [2].

### 2.6 Quantitative RT-PCR

Total RNA was extracted using the RNeasy Kit (Qiagen), and polysomal RNA was extracted with the Direct-zol™ RNA Kit (Zymo Research) according to the manufacturer's instructions. For complementary DNA (cDNA) synthesis, oligo-dT primers and the IMPROM II Reverse Transcriptase Kit (Promega) were used according to the manufacturer's instructions. Quantitative RT-PCR (RT-qPCR) was performed by using a SYBR green PCR premixture (Applied Biosystems - Foster City, CA, EUA). Normalization was performed using the internal control GAPDH (glyceraldehyde phosphate dehydrogenase), and all reactions were performed in technical triplicate. The primers used for RT-qPCR are listed in Table 5.

**Table 5:** RT-qPCR primer sequences. Oligonucleotide primers used to analyze the differential expression of genes after 24 h of adipogenic and osteogenic differentiation.

Gene Name	Forward Primer Sequence 5′-3′	Reverse Primer Sequence 5′-3′	bp
ADH1B	GGTGGCTGTAGGAATCTGTCACAC	GGGTCCCCCGAGGATTGCCT	248
MT1E	CCTGCTGCCCCATGAGCTGT	CCGGACATCAGGCACAGCAGC	90
FADS2	CAAAAGCCGAAAGCGAAGAGG	CCCACGAATTCCAGGTCAGG	368
VEGFA	CTACCTCCACCATGCCAAGTG	TGCGCTGATAGACATCCATGA	101
WNT2	TGAACGCCCCTCTCGGTGGA	GCCCTGGCTAATGGCACGCA	203
CRABP2	AGAGAACCTGACGACCCGGCG	TCCACTGCTGGCTTGGACGC	232
GAPDH	GGCGATGCTGGCGCTGAGTAC	TGGTTCACACCCATGACGA	149
